# Outcomes of severely ill patients with AIDS treated with efavirenz or dolutegravir: a multicenter, observational study

**DOI:** 10.3389/fmed.2024.1302710

**Published:** 2024-02-28

**Authors:** Carlos Brites, Marcus Lacerda, Eduardo Sprinz, Monica Bay, Gustavo Pinto, Pollyanna Azevedo, Estela Luz, Liliane Lins-Kusterer, Eduardo M. Netto

**Affiliations:** ^1^Laboratório de Pesquisa em Infectologia, Department of Medicine, Hospital Universitário Professor Edgard Santos - EBSERH, Federal University of Bahia, Salvador, Brazil; ^2^Fundação de Medicina Tropical de Manaus, Manaus, Brazil; ^3^Hospital de Clínicas de Porto Alegre, Federal University of Rio Grande do Sul, Porto Alegre, Brazil; ^4^Federal University of Rio Grande do Norte, Natal, Brazil; ^5^Federal University of Santa Catarina, Florianópolis, Brazil

**Keywords:** early mortality, advanced AIDS, dolutegravir, efavirenz, adverse (side) effects

## Abstract

**Background:**

Currently, integrase inhibitors (INIs)-based ART regimens are the preferred initial therapy for AIDS patients. There is scarce information on the use of dolutegravir (DTG) among late-presenter people living with HIV (PLHIV).

**Objectives:**

To compare the effect of DTG- or efavirenz (EFV)-based regimens on the outcomes of patients with advanced AIDS.

**Methods:**

We compared two cohorts of consecutive symptomatic AIDS patients (WHO stage 4, CD4 count<50 cells/mL) starting therapy with DTG-based (2018–2021, prospective cohort) or EFV-based regimens (2013–2016, retrospective cohort) from five Brazilian cities. The main endpoints were early (all-cause) mortality, viral suppression at 24 and 48 weeks, changes in CD4 count, and changes in initial therapy (for any reason).

**Results:**

We included all eligible patients in a consecutive way (in both groups) until we reached 92 individuals per arm. The median baseline CD4 count (20 vs. 21 cells/mL) and the median HIV plasma viral load (5.5 copies/mL log_10_) were identical across the groups. Viral suppression rates were higher in the DTG group than in the EFV group at 24 (67.4% vs. 42.4%,) and 48 weeks (65.2% vs. 45.7%, *p* < 0.001 for both comparisons). More patients in the DTG group presented with CD4 > 200 cells/mL compared to the EFV group at 48 weeks (45% vs. 29%, *p* = 0.03). Treatment changes (ITT, M = F) were significantly more frequent in the EFV group (1% vs. 17%, *p* < 0.0001). The relative mortality rate was 25% lower in the DTG group, but without statistical significance.

**Conclusion:**

We detected a higher rate of virological suppression and greater treatment durability in patients with advanced AIDS treated with DTG than in those treated with EFV.

## Introduction

In Brazil and other low- and middle-income countries, there is still a large proportion of people living with HIV (PLHIV) who are diagnosed with AIDS or only seek healthcare late in the course of the disease. According to the Jointed United Nations Programme on HIV/AIDS (UNAIDS) 2021 Report, in Latin America, the percentage of patients with a CD4 cell count of <200 cells/mm3 at diagnosis varies from 10% in Uruguay to 44% in Paraguay, with Brazil recording 27% of cases (1). In countries where the median of first CD4 cell count is usually below 200 cells/mm3, the problem of early mortality remains an important concern (2–4).

The early mortality rate, defined as the proportion of patients dying during the first year of therapy, is variable and can range from as low as 2% in developed countries to up to 29% in certain regions of the world (4, 5). The usual factors driving higher rates of early mortality are low baseline CD4 cell count, male sex, advanced World Health Organization (WHO) clinical stage, low body mass index, anemia, age greater than 40 years, and pre-antiretroviral therapy (ART) quantitative HIV RNA (5). Despite the high efficacy of current ART regimens for ART-naive PLHIV in clinical trials, they usually do not include patients with advanced disease, making it difficult to assess the impact of new drugs/regimens on early mortality rates.

Integrase inhibitor-based combination antiretroviral therapy (cART) is the preferred recommendation for initial treatment of HIV-infected patients according to the international guidelines on HIV treatment (6, 7). Dolutegravir (DTG) is currently recommended as a first-line treatment option by the World Health Organization, regardless of sex, pregnancy status, or stage of disease (8). DTG-based cART has been shown to be superior to alternative regimens based on efavirenz (EFV) or ritonavir-boosted darunavir for patients starting therapy. Furthermore, DTG-based cART promotes faster viral suppression compared to other options (9, 10). However, the available evidence on its efficacy in the treatment of ART-naive PLHIV relies on clinical trials that mostly included patients with CD4+ cell count higher than 200 cells/mm^3^. There is scarce information on the use of DTG for the treatment of advanced disease in late-presenter PLHIV.

In Brazil, DTG is the recommended first-line drug for patients starting antiretroviral therapy since 2017. Data from the Brazilian Ministry of Health demonstrated that DTG was superior to ritonavir-boosted LPV- or EFV-based regimens in patients starting ART, in a real-world experience (11). However, the study included all patients starting therapy and did not provide any information on their clinical stage of infection. We aimed to compare the effect of DTG- or EFV-based regimens on early mortality rates, frequency of adverse events, CD4 changes from baseline, and treatment changes/discontinuation in patients with advanced AIDS (WHO classification stage 4 and baseline CD4 cell count below 50 cells/mm^3^).

## Methods

### Study design and participants

This observational, ambispective cohort included patients starting cART from five large Brazilian cities (Salvador, Natal, Manaus, Florianópolis, and Porto Alegre). All sites were main public referral centers for AIDS care, with well-trained healthcare professionals in HIV management. Clinical and laboratory data were recorded at baseline, 24 weeks, and 48 weeks after enrollment. Data collected during unsolicited visits due to various reasons such as the onset of adverse events, change of therapy, or other outcomes of interest were also recorded.

### Inclusion criteria

ART-naive patients starting ART from 2018 to 2020 were included if they met the following criteria: an HIV-1 RNA plasma viral load >1,000 copies/mL, a WHO clinical stage 4, a baseline CD4 count below 50 cells/mm3, and were at least 18 years of age at the time of enrollment. They started a lamivudine/tenofovir (fixed-dose combination) plus DTG regimen. A second, retrospective cohort was used for comparison purposes. It included patients with a similar profile who started therapy from 2013 to 2016, at each site, with a fixed-dose combination of lamivudine plus tenofovir and EFV regimen. Data from the retrospective cohort were obtained by reviewing medical charts. Either DTG- or EFV-based regimens were the recommended therapy for ART-naive patients during their respective study periods, according to the Brazilian guidelines for antiretroviral therapy. Patients in both groups were included consecutively, starting with the first eligible subjects who attended during the study period, until the sample was completed.

### Procedures

We documented the number and causes of deaths during the study period. The main outcome for comparison was early mortality rates, defined as all-cause mortality at 1 year from ART initiation and any cause of interruption of therapy, including death, changing ART, and lost to follow-up (FU). The secondary main outcome was the proportion of patients with a plasma HIV-1 RNA viral load (VL) of less than 50 copies per mL at week 48. Virological failure was defined as a VL > 200 copies/mL. Additional secondary outcomes at week 48 were changes in the CD4 cell count (the proportion of patients achieving a CD4 count higher than 200 cells per mL) and the proportion of patients changing initial therapy for any reason.

The baseline date was defined as the date when patients started ART. The HIV route of transmission was categorized as persons who inject drugs (PWID), men who have sex with men (MSM), occupational exposure, and persons whose only self-reported risk was heterosexual contact. The predictor variables were sex, age, baseline CD4 cell count, and HIV-1 RNA plasma VL. Additional laboratory variables included white blood cells, hemoglobin, platelets, liver enzymes, creatinine, fasting glucose, and serum lipids (fasting total cholesterol, LDL, HDL, and triglycerides). All laboratory tests were performed at baseline, 24 weeks, and 48 weeks.

The study was approved by the ethics committees of all participant institutions and was conducted in accordance with the principles of the Helsinki Declaration. All participants in the prospective cohort provided written informed consent.

### Statistical analysis

**Figure 1:**
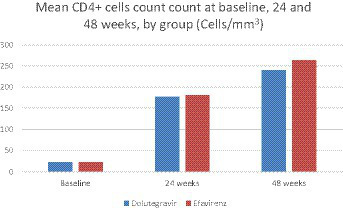
Mean CD4 cell count at baseline, 24 weeks, and 48 weeks, per group.

For sample size calculation, we used a previous study conducted at a referral hospital in Salvador, which showed an early mortality rate of 33% for patients admitted with advanced AIDS and treated with EFV (12). Considering that the estimated mortality in the AIDS clinics of our hospital was much lower (14%) for a similar population, in recent years, we hypothesized that patients treated with DTG since 2018 would have a mortality rate of 13%. This resulted in a sample size of 184 participants (92 per group) using the Fleiss proposed test with continuity correction, with a 95% CI and 80% power (hazard ratio equal to 2.5). The significance was calculated for each time point in Figure 1. The Wilson score method was used to calculate the confidence interval for proportions. For each table, the confidence intervals were adjusted for multiple comparisons with a Bonferroni correction.

The difference in proportions for nominal variables and treatment groups was assessed by the Pearson chi-squared test. The Mann–Whitney U test was used to compare continuous variables. Viral suppression rates (VL < 50 copies/mL) at 24 and 48 weeks were defined by the US Food and Drug Administration (FDA) snapshot algorithm in the intention-to-treat population (ITT, M = F). Kaplan–Meier survival curves were used to compare the study groups. The composite primary outcomes consisted of death (any cause), therapy change/discontinuation, failure to achieve virological suppression, and lost to follow-up. The log-rank test was used to compare the occurrence of either therapy interruption/changes or death over time. All statistical analyses were performed using SPSS version 25. The significance level was set at 0.05.

## Results

Between 2018 and 2020, a total of 92 eligible patients who started DTG-based ART were enrolled in the study across five HIV referral sites in large Brazilian cities. The medical charts of an equal number of subjects who started therapy with EFV-based regimens between 2013 and 2016 were reviewed. All eligible patients in the study period were invited to participate until the desired sample size was achieved. Table 1 shows the main characteristics of the patients included in the study. In both groups, two-thirds of subjects were men and were infected by HIV through sexual exposure.

**Table 1:** Sociodemographic characteristics and comorbidities of patients, by group.

	Group		*p*
	Dolutegravir	Efavirenz	
Age—median (IQR)	38.6 (31.1–44.5)	35.43 (31.4–41.6)	0.172
Age greater than 40 years	39 (42.4)	31 (33.7)	0.224
Male sex—*n* (%)	63 (68.5)	59 (64.1)	0.533
Self-reported transmission—*n* (%)	39 (42.9)	31 (33.7)	<0.001
Heterosexual	54 (58.7)	23 (25.0)	
Bi/Homosexual	30 (32.6)	9 (9.8)	
Occupational	0 (0)	1 (1.1)	
Unknown	8 (8.7)	59 (64.1)	
Smoking			<0.001
Current	17 (18.5)	18 (19.6)	
Past	15 (16.3)	10 (10.9)	
Never smoked	60 (65.2)	35 (38.0)	
NA	0 (0)	29 (31.5)	
Illicit drug use			<0.001
Yes	13 (14.1)	11 (12.0)	
No	79 (85.9)	44 (47.8)	
NA	0 (0.0)	37 (40.2)	
Anemia			0.015
Yes	39 (42.4)	34 (37.0)	
No	53 (57.6)	50 (54.3)	
NA	0 (0.0)	8 (8.7)	
Diabetes mellitus	6 (6.5)	1 (1.1)	0.054
Arterial hypertension	10 (10.9)	5 (5.4)	0.178

MSM, men who have sex with men; NA, not available.

At baseline, the median viral load was 5.5 (IQR: 5.05–5.91) and 5.65 log_10_ RNA copies/mL (IQR: 5.10–5.98) for DTG and EFV groups, respectively (*p* = 0.86). The mean CD4 cell count was also similar for DTG (22 cells/mm3, 95% CI: 19–25) and EFV groups (23 cells/mm3, 95% CI: 20–26, *p* = 0.94). All patients presented with stage 4 according to the WHO classification. Although the distribution of opportunistic infections was similar for both groups, the combined frequency of neurotoxoplasmosis and *Pneumocystis jirovecii* pneumonia was 2.1 times higher in the DTG group than in the EFV group (51 vs. 24 for DTG and EFV groups, respectively, *p* < 0.001). Patients diagnosed with active tuberculosis were treated with DTG bid, as recommended by the international guidelines for HIV treatment. No cases of Immune Reconstitution Inflammatory Syndrome (IRIS) were reported during the study.

**Figure 2:**
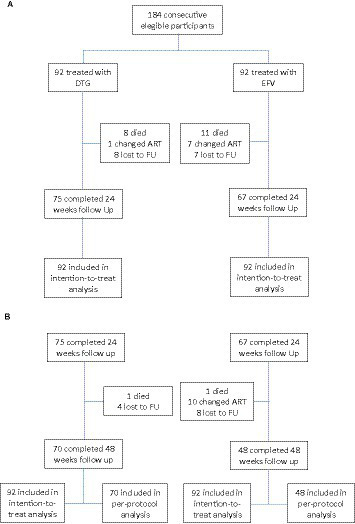
Study profile. **(A)** Patients’ disposition at 24 weeks. **(B)** Patients’ disposition at 48 weeks.

After 24 weeks of follow-up, 62 (67.4%) patients in the DTG group had a plasma viral load <50 copies/l, vs. 39 (42.4%) in the EFV group (*p* < 0.001). At week 48, 63 (68.5%) patients in the DTG group had a plasma viral load below 50 copies/mL, compared to 40 (43.5%) patients in the EFV group (*p* = 0.001). Mean CD4 cell count was similar across groups at week 24 (144 ± 116 cells/mm3, 95% CI: 120–168, and 133 ± 117 cells/mm3, 95% CI: 98–167, for DTG and EFV groups, respectively, *p* = 0.1), and week 48 (240 ± 104 cells/mm3, 95% CI: 225–211 and 262 ± 151 cells/mL, 95% CI: 208–300, for DTG and EFV groups, respectively, *p* = 0.6). After 48 weeks, 41 (44.6%) patients in the DTG group reached a CD4 cell count >200 cells/mm3, vs. 27 (29.3%) in the EFV group (*p* = 0.03, ITT). Figure 2 shows the study profile, while Figure 1 shows the variation of mean CD4 cell count per group from baseline to 24 and 48 weeks of treatment.

Table 2 shows the most frequent diagnosis presented by patients at their first medical visit. The frequency of AIDS-related conditions was similar for both groups.

**Table 2:** Most frequent AIDS-related conditions at baseline, by group.

	Study group							
	Dolutegravir (*n* = 92)				Efavirenz (*n* = 92)			
	*n*	%	95%CI		*n*	%	95%CI	
Esophageal candidiasis	28	30.8%	22.0%	−40.7%	25	27.5%	19.1%	−37.2%
Neurotoxoplasmosis	30	32.6%	23.7%	−42.6%	12	13.0%	7.3%	−21.0%
P. jirovecii pneumonia	21	22.8%	15.2%	−32.2%	14	15.2%	9.0%	−23.6%
Bacterial pneumonia (recurrent)	15	16.3%	9.8%	−24.8%	14	15.2%	9.0%	−23.6%
Tuberculosis	14	15.2%	9.0%	−23.6%	16	17.4%	10.7%	−26.1%
Chronic diarrhea	10	10.9%	5.7%	−18.4%	15	16.3%	9.8%	−24.8%
Wasting syndrome	9	9.8%	5.0%	−17.1%	9	9.8%	5.0%	−17.1%
Neurocryptococcosis	7	7.6%	3.5%	−14.4%	3	3.3%	0.9%	−8.4%
Cytomegalovirus infection	5	5.4%	2.1%	−11.5%	1	1.1%	0.1%	−5.0%
Disseminated histoplasmosis	5	5.4%	2.1%	−11.5%	3	3.3%	0.9%	−8.4%
Malignancies	4	4.3%	1.5%	−10.0%	2	2.2%	0.5%	−6.8%
Disseminated herpes Zoster	4	4.3%	1.5%	−10.0%	0	0.0%		

The only significant differences observed in laboratory results at 48 weeks were in mean levels of triglycerides (mean 128 ± 64.5 mg//dL in the DTG group versus 187.5 mg/dL in the EFV group, *p* < 0.01), total cholesterol (167.9 ± 36.5 mg/dL in the DTG group vs.188.8 ± 50 mg/dL in the EFV group, *p* = 0.04), VLDL cholesterol (26.2 ± 13.5 mg/dL vs. 35.2 ± 18.0 mg/dL in the EFV group, *p* = 0.01), and creatinine (1.0 ± 0.2 mg/dL in DTG group vs. 0.9 ± 0.2 mg/dL in the EFV group, *p* = 0.02). Liver enzymes, platelet count, glucose, and hemoglobin levels did not differ across groups at week 48. Table 3 shows the laboratory results at baseline and after 48 weeks.

**Table 3:** Laboratory results at baseline and 48 weeks.

		Group			Group					
		Dolutegravir		Efavirenz	Dolutegravir				Efavirenz	
	*N*	Mean (SD)	N	Mean (SD)	*p* value	N	Mean (SD)	*N*	Mean (SD)	*p* value
Hematocrit	92	31.3 (6.6)	79	31.6 (6.4)	0.75	68	41.3 (5.4)	37	40.7 (3.4)	0.49
Leucocytes	92	5.305(3463)	83	4.821 (3,133)	0.34	67	5,448 (1,375)	39	5,705 (1,927)	0.43
Platelets	92	237.157 (119.275)	84	246,407 (123,674)	0.61	67	232,700 (68,044)	38	227,716 (80,742)	0.74
Creatinine	92	0.95 (0.62)	76	0.98 (1.39)	0.85	65	0.97 (0.22)	33	0.86 (0.19)	0.02
Glucose	86	99.7 (23.8)	50	92.6 (21)	0.08	63	95.3 (21.9)	31	91.4 (20.9)	0.41
AST	79	168.0 (101)	40	180.6 (84.5)	0.50	66	128.5 (64.5)	29	187.5 (116.8)	0.02
ALT	92	102.1 (474.2)	81	74.2 (113.9)	0.61	68	28.5 (12.9)	36	29.7 (16.5)	0.69
Cholesterol total	92	77.8 (145.7)	80	76.0 (118.8)	0.93	68	30.6 (19.9)	36	30.6 (19.3)	0.99
HDL	80	159.6 (50.6)	40	167.9 (109.6)	0.57	64	167.9 (36.5)	32	188.7 (50)	0.04
LDL	79	33.7 (15.1)	40	26.4 (13.7)	0.01	64	45.0 (31.8)	32	44.8 (17.9)	0.98
Triglycerides	75	93.5 (36.5)	39	90.8 (40.5)	0.72	62	103.2 (32.8)	26	110.9 (39.9)	0.35
VLDL	75	32;7 (18.3)	36	37.9 (16.3)	0.15	63	26.2 (13.5)	26	35.2 (18)	0.01
Alkaline Phosphatase	86	294.9 (312.9)	56	347.9 (488.7)	0.43	61	203.9 (129.9)	14	256.1 (138.2)	0.18

Table 4 summarizes the results of viral load, CD4 cell count, and other outcomes during the study period. The follow-up was completed by 70 (76.1%) patients in the DTG group and by 48 (52.2%) patients in the EFV group at 48 weeks. Nine (9.8%) patients in the DTG group and 12 (13%) patients in the EFV group died during the first 48 weeks of therapy. The most frequent cause of death was bacterial infection, as shown in Table 5. The causes of death were similar across groups. The mean elapsed time between starting therapy and death was similar for the DTG (66.1 ± 62.4 days) and EFV (63.5 ± 55.9 days, *p* = 0.9) groups. Only five deaths occurred after 100 days of treatment.

**Table 4:** Patients’ outcomes at 48 weeks, according to treatment group.

	Study group							
	Dolutegravir				Efavirenz			
	*n*	%	95%CI		*n*	%	95%CI	
Viral load <200 copies/mL	68	73.9%	64.3%	−82.0%	44	49.4%	39.2%	−59.7%
Viral load <50 copies/mL	63	68.5%	58.5%	−77.3%	40	44.4%	34.5%	−54.8%
Viral load <50 copies/mL (per protocol)	63	90.0%	81.4%	−95.4%	40	83.3%	71.0%	−91.8%
Deaths	9	9.8%	5.0%	−17.1%	12	13.0%	7.3%	−21.0%
ART modification	1	1.1%	0.1%	−5.0%	17	18.5%	11.6%	−27.3%
Lost to follow-up	12	13.0%	7.3%	−21.0%	15	16.3%	9.8%	−24.8%
CD4 > 200 cells/mm3	41	44.6%	34.7%	−54.8%	27	29.3%	20.8%	−39.2%

^*^p < 0.001, Pearson chi-square test (ITT, M-F).

**Table 5:** Frequency and causes of death during the study period, by group.

Cause of death	DTG *N* (%)	EFV *N* (%)	Total (%)
Bacterial infection	3 (33.3)	5 (41.7)	8 (38.1)
Neurotoxoplasmosis	4 (44.4)	1 (8.3)	5 (23.8)
*P. jirovecii* pneumonia	1 (11.1)	0	1 (4.8)
Disseminated strongyloidiasis	1 (11.1)	0	1 (4.8)
Central nervous system lymphoma	0	1 (8.3)	1 (4.8)
Metastatic anal cancer	0	1 (8.3)	1 (4.8)
Disseminated histoplasmosis	0	1 (8.3)	1 (4.8)
Disseminated tuberculosis	0	1 (8.3)	1 (4.8)
Neurosyphilis	0	1 (8.3)	1 (4.8)
Neurotuberculosis	0	1 (8.3)	1 (4.8)
Anaphylactic shock	0	1 (8.3)	1 (4.8)
Total	9 (9.8)	12 (13.0)	21 (11.4)

**Figure 3:**
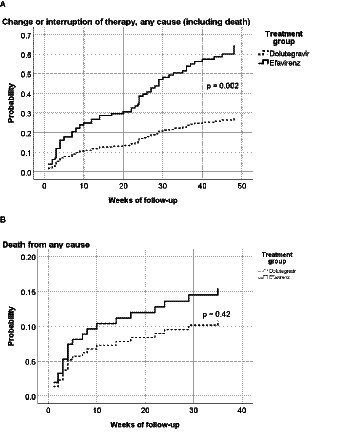
Hazard ratio for any-cause interruption of therapy (including death) **(A)** or any-cause death **(B)**, by treatment group. *p* = 0.002 in the log-rank (Mantel-Cox) test; Hazard ratio = 0.44 (95%CI: 0.26–0.73). *p* = 0.42 in the log-rank (Mantel-Cox) test; Hazard ratio = 0.70 (95%CI: 0.30–1.66).

Furthermore, 12 (13.0%) and 16 (16.3%) patients were lost to follow-up in the DTG and EFV groups, respectively. The proportion of patients changing/discontinuing therapy was significantly higher in the EFV group (16.3%) than in the DTG group (1.1%, *p* < 0.001). The main reason (7 out of 18 patients) for changing therapy was central nervous system adverse events, followed by virological failure (4 out of 18 patients) and rash (2 cases). The only patient who changed therapy in the DTG group was diagnosed with progressive multifocal leukoencephalopathy only 3 weeks after ART initiation, and his attending physician decided to replace DTG with ritonavir-boosted darunavir based on the personal experience with this regimen in such situations. A significantly higher proportion of patients in the EFV group had changed/interrupted ART at week 48, as shown in Figure 3 (*p* = 0.002).

Three fewer deaths were reported in the DTG group in comparison to the EFV group. The difference between the EFV (13%) and DTG (9.8%) groups was 3.2%, representing a 25% relative decrease in deaths in the DTG group.

## Discussion

Patients with advanced AIDS who received the DTG-based ART regimen experienced a lower likelihood of treatment interruption/change and higher rates of virological suppression after 48 weeks of treatment compared to patients with a similar profile who were treated with an EFV-based ART regimen. In addition, patients treated with the DTG-based regimen had a higher likelihood of achieving a CD4 cell count higher than 200 cells/mL after 48 weeks. In the present study, the early mortality rate was higher among patients receiving EFV; however, the difference was not statistically significant.

Viral load suppression (<50 copies) rates were significantly higher in the DTG group after 24 and 48 weeks. In addition, significantly more patients in the DTG group reached a CD4 cell count higher than 200 cells/mL after 48 weeks. Only one patient changed therapy in the DTG arm (because of physician choice), compared with 17 in the EFV arm (all due to adverse events). The SINGLE study demonstrated a higher efficacy of DTG-based regimens compared with EFV-based regimes (9). However, the trial included fewer than 10% of patients with a CD4 count lower than 200 cells/mL. The current work demonstrates that DTG-based regimens are as effective for the treatment of patients with advanced AIDS as those starting therapy in an earlier phase of the disease.

The early mortality rate was similar for both groups. However, although not reaching statistical significance, the relative mortality difference was 25% lower in the DTG group than in the EFV group. Most of the causes of death were infections, especially bacterial ones, and AIDS-defining illnesses. A high proportion of deaths occurred in the first months of therapy, caused by AIDS-associated conditions. Most of the deaths (13/21) occurred in the first 2 months of therapy and were caused by conditions diagnosed at baseline. Only five deaths occurred after 100 days of treatment. This reinforces the severity of patients’ diseases at presentation and the limited effectiveness of cART in modifying such outcomes. In the REALITY trial, the mortality rate in patients with a similar profile to those participants in the present study was as high as 25%, with 18% dying before week 12. In addition, women were significantly less likely to die in the REALITY trial, a finding also detected in our study (13).

A potential explanation for the relative lower mortality rates in the DTG group is the better immune recovery presented by patients in the prospective cohort. Although the two cohorts were treated in different time periods, the only change in the standard of care for PLHIV between 2013–2016 and 2018–2021 was the replacement of EFV by DTG as first-line therapy. In addition, the similar median CD4 count, median viral load, and frequency of opportunistic conditions at baseline demonstrate that groups were comparable, and no important selection bias was detected. Although faster viral suppression is a characteristic of integrase inhibitor-based treatment, there is no previous evidence showing a clear immunological benefit of it. However, recent studies showed that low-level viremia as well as viremic time are predictive of mortality in patients with HIV (14, 15). Our findings suggest that severely ill patients can benefit from faster virological suppression.

The safety profile of both drugs was significantly different, with more patients changing therapy due to the onset of adverse events in the EFV group. As expected, central nervous system-related adverse events were the most frequent cause of therapy change in the EFV group. In the NAMSAL study, the frequency of adverse events was similar for patients using DTG or a lower dose (400 mg) of EFV (16). A similar result was observed in the ADVANCE study that compared two DTG-based regimens (containing tenofovir disoproxil or tenofovir alafenamide) versus efavirenz plus tenofovir and emtricitabine (17). Repeatedly, most of the patients included in these trials had a higher baseline CD4 cell count than those included in the present study.

The difference in laboratory results for both groups after 48 weeks was limited to cholesterol/triglycerides changes that were significantly higher in the EFV group, while mean creatinine levels were slightly higher in the DTG group. However, the detected changes were not clinically relevant and were in accordance with the safety profiles of both drugs observed in previous studies.

The proportion of patients lost to follow-up was higher (15.2%) in the EFV group compared to the DTG group (10.9%); however, the difference did not reach statistical significance. Although moving to another city or other referral center for HIV is a common reason for not attending the scheduled evaluations, missed clinic visits are independently associated with all-cause mortality (18, 19). In Brazil, retention in care at 12 months remains a problem, with a loss of 15% of patients initiating ART (according to the Brazilian Ministry of Health), and the absence of a reliable way to trace them when they do not attend the visits is a main challenge (20).

Despite the advances in HIV treatment, in Brazil, 27% of patients had a late diagnosis of HIV infection in 2021 (CD4 < 200 cells/mm^3^) (1, 20). Among patients starting ART in 2020, only 78% had an adequate adherence to therapy, and 9% were lost to follow-up. Early access to care, higher educational level, and higher income were factors predictive for better retention to care in a referral HIV center in Rio de Janeiro (21). Another study in the same setting showed that the level of early retention in care was lower for patients with lower socioeconomic conditions and advanced disease (22). The lost-to-follow-up rate was 20%, and it was associated with an overall early mortality rate of 8%. Although such rates are like those detected in the present study, the cited study included all patients attended in the referral site, with a large proportion of them engaging with care at earlier stages of HIV infection.

Our study has some limitations, such as the use of historical controls (which is a potential risk of bias) and the small sample size. Moreover, the retrospective collection of adverse events could not accurately reflect the real incidence/severity of them. However, we had two comparable groups of patients, from several Brazilian regions, with similar clinical and immunological characteristics at entry, who were attended in referral centers for AIDS care, reducing the risk of selection bias. In addition, we used the standard of care for PLHIV recommended by the Brazilian guidelines during the two study periods (23). Due to the changes in the Brazilian recommendations for antiretroviral therapy in recent years, it would not be possible to use a contemporary control group. The small sample size could also limit our capacity to detect mortality differences; however, due to the exploratory design of the study and the difficulties in recruiting patients with advanced disease, it was not possible to expand the number of included patients.

Our findings demonstrate that a DTG-based regimen is more effective than the regimen based on EFV for the treatment of severely ill patients with AIDS, with a higher rate of virological suppression, higher treatment durability, and better immune restoration. Although the higher frequency of neurotoxoplasmosis and *P. jirovecii* pneumonia (two severe opportunistic infections) in the DTG group could increase the mortality rate in this group, no such occurrences were reported in this group. This reinforces the current recommendations of most guidelines for the use of DTG-based regimens as first-line therapy for ART-naive AIDS patients, especially for those with advanced disease.

## Data availability statement

The datasets presented in this study can be found in online repositories. The names of the repository/repositories and accession number(s) can be found at: https://fbai.org.br.

## Ethics statement

The studies involving humans were approved by Comitê de ètic em Pesquisa da Maternidade Climerio de Oliveira - Universidade Federal da Bahia. The studies were conducted in accordance with the local legislation and institutional requirements. The participants provided their written informed consent to participate in this study.

## Author contributions

CB: Conceptualization, Funding acquisition, Investigation, Methodology, Project administration, Supervision, Validation, Writing – original draft, Writing – review & editing. ML: Investigation, Supervision, Validation, Writing – review & editing. ES: Investigation, Supervision, Validation, Writing – review & editing. MB: Investigation, Supervision, Validation, Writing – review & editing. GP: Investigation, Validation, Writing – review & editing. PA: Investigation, Validation, Writing – review & editing. EL: Data curation, Investigation, Supervision, Validation, Writing – review & editing. LL-K: Formal analysis, Validation, Writing – review & editing. EN: Formal analysis, Methodology, Validation, Writing – review & editing.
